# Supplementary material for: The mechanics of landslide mobility with erosion
Source: Nat Commun. 2021 Nov 23;12:6793. doi: 10.1038/s41467-021-26959-5 (PMC8611095; doi:10.1038/s41467-021-26959-5)
Supplement: Supplementary file 1 — Supplementary Information [file 41467_2021_26959_MOESM1_ESM.pdf]

The Mechanics of Landslide Mobility with Erosion

Shiva P. Pudasaini [a,b,*], Michael Krautblatter [a]

[a] Technical University of Munich, Chair of Landslide Research
Arcisstrasse 21, D-80333, Munich, Germany

[b] University of Bonn, Institute of Geosciences, Geophysics Section
Meckenheimer Allee 176, D-53115, Bonn, Germany

*Corresponding Author, E-mail: shiva.pudasaini@tum.de

Supplementary Information

Supplementary Note 1.

Correct Derivation of the Relevant Momentum Balance Equation: Elegant Formulations. Here, we derive a basic erosional landslide equation in the most elegant way without any condition. Much more intuitive derivation has been presented in the paper. The situation of an erosive landslide is as follows. Let $m + (-\Delta m)$ be the mass of the landslide that moves with velocity u at time $t_1 = t$. Then, at time $t_2 = t + \Delta t$, after entraining the mass Δm, the actual landslide of mass m moves with velocity $u + \Delta u$, and the other portion of the mass $(-\Delta m)$ moves with the erosion velocity u^b.

So, the momentum P_1 of the landslide at time t_1, and the momentum P_2 of the landslide and the eroded mass at time t_2, respectively, are:

$$P_1 = [m + (-\Delta m)]\, u, \tag{S.1}$$

and

$$P_2 = [m + (-\Delta m) + \Delta m]\,(u + \Delta u) + (-\Delta m)\, u^b = m\,(u + \Delta u) + (-\Delta m)\, u^b. \tag{S.2}$$

The form of P_2, particularly the appearance of the negative sign in $(-\Delta m)u^b$ in (S.2), may seem to be abnormal. In fact, the negative sign is not due to the negative change of the mass but rather due to the component $(-\Delta m)$ in the mass at time t_1. This is consistent with the momentum conservation for the erosional landslide. We make this clearer now. This can be proven rigorously in many different but equivalent ways. We present here one of the best ways: In time t_2 the mass $(m - \Delta m)$ moves with velocity $(u + \Delta u)$, and the entrained mass Δm moves with the velocity $(u + \Delta u) - u^b$. These two components in P_2 can be re-arranged as follows:

$$\begin{aligned} P_2 &= (m - \Delta m)(u + \Delta u) + \Delta m \left[(u + \Delta u) - u^b\right] = [(m - \Delta m)(u + \Delta u) + \Delta m(u + \Delta u)] + \Delta m(-u^b) \\ &= m(u + \Delta u) + \Delta m \left(-u^b\right) = m(u + \Delta u) + (-\Delta m)\, u^b. \end{aligned} \tag{S.3}$$

So, looking on the nicely selected form of the momentum $P_1 = [m + (-\Delta m)]u$ at t_1, we can legitimately separate the mass into two elements m and $(-\Delta m)$ and assign them respectively to the velocities $(u + \Delta u)$ and u^b for the text time t_2 to obtain P_2 in (S.2). This shows the graceful consideration in deriving (S.2).

Conservation of linear momentum states the following relation incorporating all the forces F including the forces applied to the landslide and the entrained mass:

$$F = \lim_{\Delta t \to 0} \frac{P_2 - P_1}{\Delta t}. \tag{S.4}$$

Since $P_2 - P_1 = m\Delta u + \left(u - u^b\right)\Delta m$, we have now the formally and correctly derived momentum equation for an erosional landslide:

$$F = m\frac{du}{dt} + u^{ev}\frac{dm}{dt}, \tag{S.5}$$

where, $u^{ev} = u - u^b$ is the entrainmnet-velocity. Moreover, dm/dt is positive. We call (S.5) the (basic) erosional landslide equation. Equation (S.5) can be obtained in many different ways. One may derive a similar equation for the depositional landslide.

All three derivations, one in the paper and two presented above, lead to the same result. This proves that we are physically and mathematically fully consistent.

It is important to note that $P_2 - P_1 = m\Delta u + \left(u - u^b\right)\Delta m$ is the main structure that any physically correct derivation must produce for the erosional landslide. This is clear from the two alternative derivations presented above, and another in the paper. Moreover, at this point, it is crucial to realize, that the momentum equation (S.5) for the erosional landslide must be derived rigorously as done here by following the first-principles (also see the paper), but cannot just be speculated arbitrarily.

We call (S.5) the landslide-rocket-equation. In the form, (S.5) is similar to the famous Tsiolkovsky Rocket-Equation[64](also see the main text). However, there are fundamental differences. First, the way we derive the model is different. We elegantly considered the mass in time t that has legitimately been split into the landslide mass minus the mass that will later be added into the landslide as the eroded mass at time $t + \Delta t$. This was vital. Second, the mass of the rocket is decreasing (since it consumes fuel), so dm/dt is negative. But, for erosional landslide dm/dt is positive as the mass of landslide is increasing. Third, although the multiplier of dm/dt is positive for both the erosional landslide and the rocket, they have quite different perspectives and mechanisms. For the rocket, it is the velocity of the exhaust, say u^{ex}. But, for the erosional landslide, it is the velocity of the landslide minus the velocity of the eroded mass that is entrained by the landslide. Thus, depending on the magnitude of the erosion velocity, the entrainment velocity u^{ev} can be substantially less than the landslide velocity, as the velocity of the eroded particle, that is entrained by the landslide, is a positive quantity that, depending on the situation (the flow and the bed morphology), can be as high as the velocity of the landslide itself.
